# Retraction: Oral leukoplakia, a clinical-histopathological study in 412 patients

**DOI:** 10.4317/jced.532746

**Published:** 2021-05-01

**Authors:** Andrea Rubert, Leticia Bagán, Jose V. Bagán

**Affiliations:** 1 Assistant Professor of Oral Medicine. European University of Valencia; 2 Associate Professor of Oral Medicine. University of Valencia. Av. de Blasco Ibáñez, 15, 46010 València; 3 Chairman of Oral Medicine. University of Valencia. Head of the Department of Stomatology and Maxillofacial Surgery University General Hospital. Valencia (Spain) Fundación de Investigación del Hospital General Universitario of Valencia, CIBERONC, Valencia, Spain

## Abstract

The authors detected some minor errors in the published manuscript (Rubert A, Bagán L, Bagán JV. Oral leukoplakia, a clinical-histopathological study in 412 patients. J Clin Exp Dent. 2020 Jun 1;12(6):e540-e546. doi: 10.4317/jced.57091. PMID: 32665812; PMCID: PMC7335600.) and have requested that the entire article be republished with these errors already rectified.

**Background:**

A retrospective clinical-histopathological study was made of the evolution of oral leukoplakia over time, staging the disease according to the classification of van der Waal.

**Material and Methods:**

A study was made of 412 patients with oral leukoplakia, analyzing the corresponding clinical factors and histopathological findings; assessing associations between the different clinical presentations and epithelial dysplasia; and evaluating the factors influencing malignant transformation of the lesions.

**Results:**

Clinically, homogeneous presentations were seen to predominate (n = 336, 81.6%), while histologically most of the lesions exhibited no dysplastic changes (n = 271; 65.7%). Stage 1 of the van der Waal classification was the most common presentation (n = 214; 51.9%). The lesion malignization rate was 8.5%, and the factors associated to a significantly increased malignization risk were non-homogeneous OL lesions (*p*=0.00), lesion location in the tongue (*p*=0.00), and the presence of epithelial dysplasia (*p*=0.00).

**Conclusions:**

In our series of patients with oral leukoplakia, malignization was associated to the less common clinical presentations of the disease, i.e., non-homogeneous lesions, and the latter tended to exhibit high grade epithelial dysplasia.

** Key words:**Oral leukoplakia, potentially malignant disorders, malignant transformation.

## Introduction

Oral leukoplakia (OL) is the most common potentially malignant lesion of the oral mucosa (1), with an estimated prevalence of 2% in the general population. The annual incidence of transformation into oral squamous cell carcinoma (OSCC) is estimated to be 1% for all types of OL (2).

Oral leukoplakia is defined as “a whitish plaque of doubtful risk after discarding other known disorders that do not pose an increased risk of cancer”. In the year 2015, van der Waal (3) defined OL as a predominantly white plaque that cannot be clinically or pathologically attributed to any other disorder. Oral leukoplakia is associated to a high risk of cancer development either in an area close to the leukoplakia lesion or in any other part of the oral cavity or head and neck region.

The prevalence of OL is reportedly higher in males between the fourth and seventh decade of life (4). In etiological terms, leukoplakia is divided into two groups: (a) idiopathic leukoplakia, in which no causal factors have been established; and (b) smoking-related leukoplakia (5). Indeed, smoking is the main established causal factor underlying these potentially malignant lesions (5,6). A synergic effect has also been reported between alcohol and smoking in relation to the development of leukoplakia and oral cancer (1).

Other described etiological factors are Sanguinaria canadensis contained in toothpastes and oral rinses, infectious agents such as *Candida*, human papillomavirus (HPV) and bacteria, nutritional and socioeconomic factors, and certain systemic disorders (6).

From the clinical perspective, two types of leukoplakia have been established: homogeneous and non-homogeneous (7). A biopsy with histopathological evaluation is required in order to establish the definitive diagnosis (8). Recent guidelines recommend differentiation between low and high risk lesions.

Although many treatment strategies have been proposed, there is no consensus regarding the best management option for OL (7). No concrete treatment has been shown to effectively prevent recurrences or the possible future development of OSCC (9). Surgical removal (conventional or laser-based) of the lesions is advised, with subsequent follow-up.

There is a some agreement that certain factors are indicative of possible malignant transformation of OL. Specifically, malignant transformation is considered to be more likely in women, in patients with long-evolving lesions, OL located on the tongue and/or floor of the mouth, lesions measuring over 200 mm2 in size, non-homogeneous lesions, and particularly OL exhibiting dysplasia in the biopsy study (10).

None of the aforementioned factors have been shown to be individually predictive of possible progression towards cancer in patients with OL. It therefore would be of interest to conduct studies involving large series of patients with OL in order to assess possible relationships among the different risk factors. In this regard, the present study was designed with the following objectives: (a) to assess possible associations between the clinical forms and the presence of epithelial dysplasia; and (b) to evaluate the evolution of the lesions after a minimum follow-up period of 5 years, and explore possible associations between the clinical forms and the presence of epithelial dysplasia and progression towards malignancy.

## Material and Methods

A retrospective clinical-histopathological study was made of 412 patients with OL diagnosed and treated in the Department of Stomatology and Maxillofacial Surgery (Valencia University General Hospital, Valencia, Spain) during the period 1994-2017. All patients met the clinical and histological conditions for establishing a firm diagnosis of leukoplakia, based on the diagnostic criteria of Warnakulasuriya *et al.* (2007) (11). In this regard, we included homogeneous leukoplakia, and among the non-homogeneous lesions we excluded proliferative verrucous leukoplakia. We also excluded cases in which the histopathological findings indicated carcinoma in situ and microinvasive carcinoma.

All patients underwent a first visit with the recording of a detailed case history, clinical exploration and photographic registry of all the lesions. A biopsy for histological study was obtained during a second visit.

The study was approved by the Ethics Committee of the University of Valencia (Valencia, Spain) (registry no. H1456655015143).

The histological results of the biopsy were recorded (no dysplasia, mild dysplasia, moderate or severe dysplasia), together with the type of treatment provided (surgery or CO2 laser vaporization at a power setting of 15 W; in the case of the latter treatment modality, the lesion was always biopsied first in order to conduct the histological study).

The outcome of the lesions after 5 years of follow-up was recorded and classified as cure, recurrence, no changes, and progression towards cancer. Of the 412 patients, 73 (17.7%) were subjected to 10 years of follow-up, while 151 patients (36.7%) were followed-up on during 5 years.

-Statistical analysis

A descriptive statistical analysis was made, with calculation of the mean, standard deviation (SD) and minimum and maximum values in the case of quantitative variables. Nonparametric tests (Kaplan-Meier survival analysis) were used to evaluate the number of patients progressing towards malignancy in each of the groups. The chi-squared test was used to assess the existence of significant differences between them. Statistical significance was considered for *p* < 0.05.

## Results

-Demographic data and habits

The mean age of our 412 patients with OL was 56.93 ± 13.76 years (range 19-89), and females (n = 281) predominated over males (n = 131). Most of the patients were non-smokers (n = 219; 53.2%) and did not consume alcohol (n = 350; 85%).

-Clinical and histopathological findings

Most of the patients (n = 327; 79.4%) were referred to our Department by a healthcare professional. The lesions were generally asymptomatic (n = 364; 88.3%).

Homogeneous leukoplakia predominated (n = 336; 81.6%), while among the non-homogeneous forms of the disease (n = 76; 18.4%) the verrucous subtype was the most common presentation (n = 39; 9.5%).

The mean lesion diameter was 18.64 ± 14.92 mm (range 1-90). In turn, the most frequently affected location was the gums (n = 168; 40.8%), followed by the tongue (n = 138; 33.5%) and the cheek mucosa (n = 130; 31.6%).

Histologically, most of the lesions (n = 271; 65.7%) showed no dysplasia, while dysplasia was observed in 141 lesions (34.3%) – with mild dysplasia being the most common presentation in the latter group (n = 98; 23.8%).

The lesions were graded based on the classification (LP) of van der Waal (2013) (3). Over half of the lesions (n = 214; 51.9%) corresponded to grade L1P0. With regard to staging of the lesions, stage 1 was the most common presentation (n = 214; 51.9%).

A total of 339 patients (82.3%) were subjected to conventional surgical treatment, while 73 (17.7%) underwent CO2 laser vaporization of the lesions.

Most of the lesions either remained without changes over follow-up (n = 164; 39.8%) or were cured (n = 160; 38.8%). Thirty-five patients (8.5%) showed progression towards malignancy (Table 1,  Table 1 cont.).

**Table 1:**
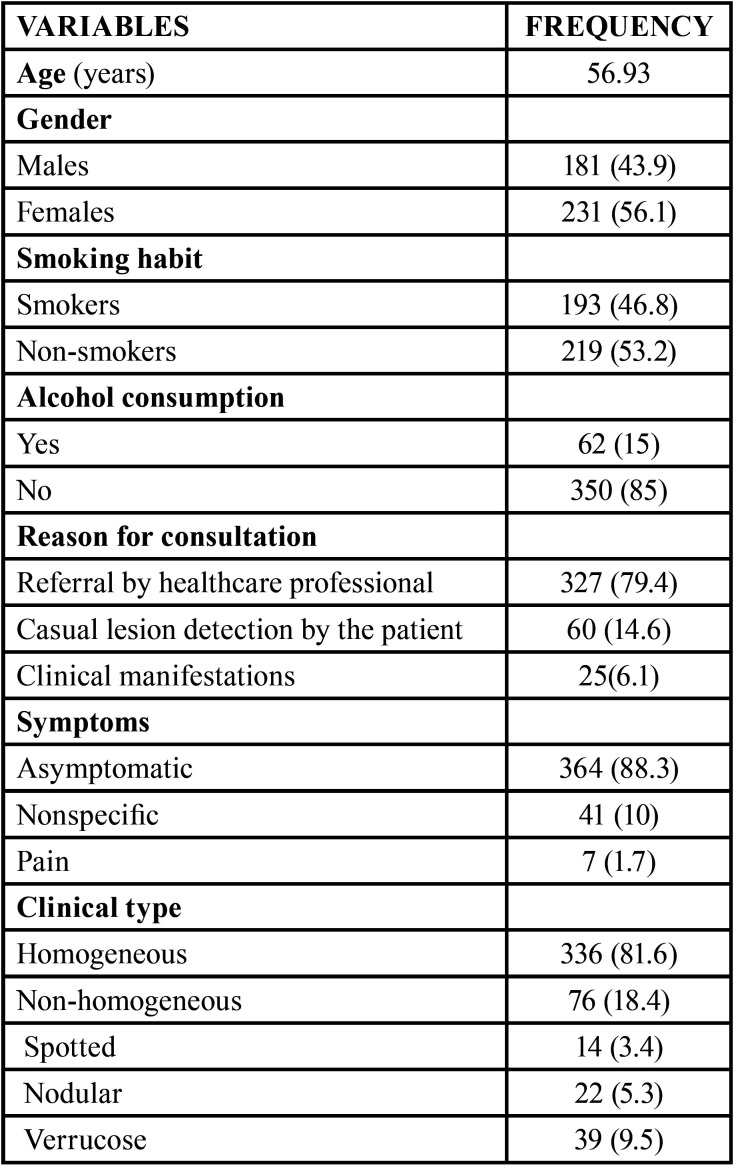
Characteristics of the patients included in the study.

**Table 1 cont.**
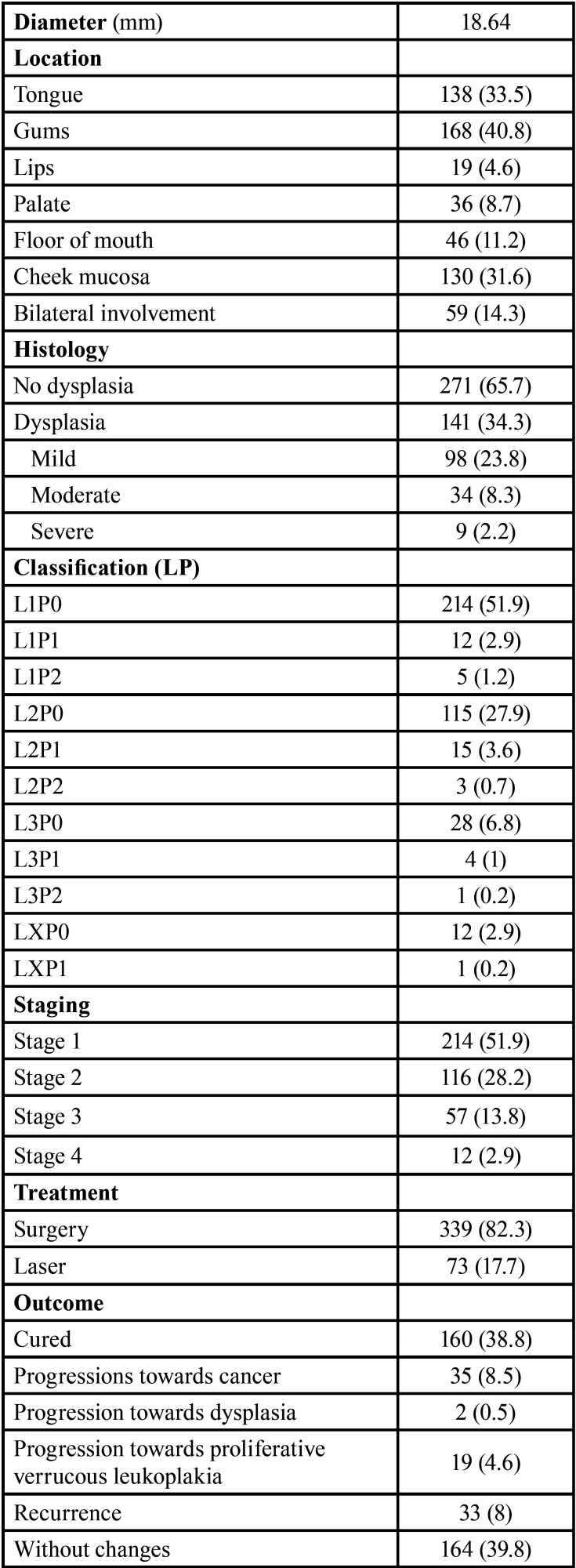
Characteristics of the patients included in the study.

Table 2 shows the results referred to the association between the clinical types of OL and patient age and gender, lesion size and location, and the histological findings.

**Table 2:**
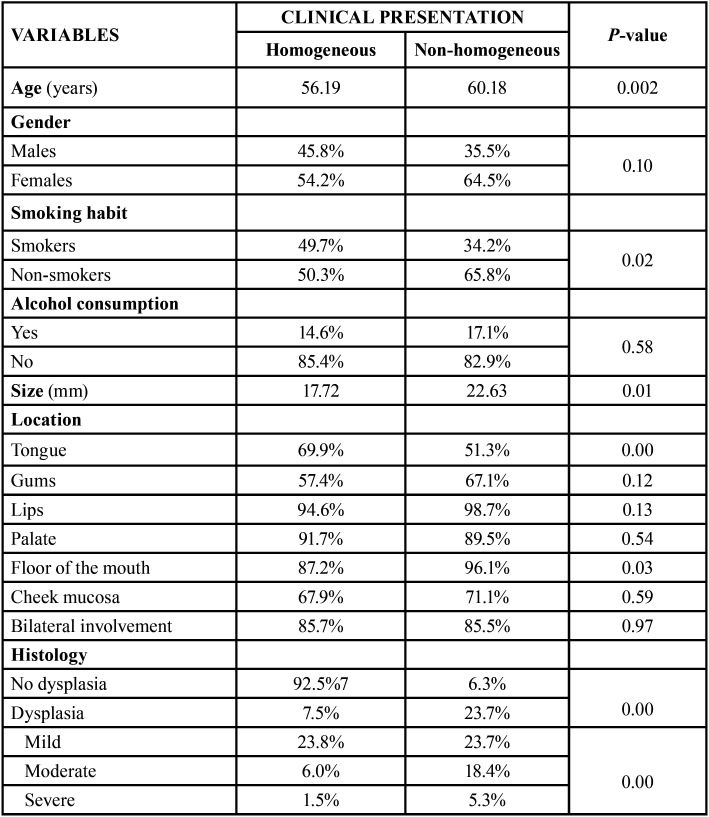
Association between the different study variables and the clinical presentation of the lesions.

The patients with clinically non-homogeneous lesions were significantly older (mean 60.18 years; *p* = 0.02). The non-homogeneous lesions were also significantly larger (mean 22.63 mm; *p*=0.01).

On the other hand, the lesions located in the floor of the mouth and tongue were the only lesions to exhibit statistically significant differences on analyzing the association between lesion location and clinical presentation – most of the lesions being non-homogeneous (*p* = 0.03) and homogeneous (*p*<0.01), respectively.

Most of the homogeneous OL lesions exhibited no epithelial dysplasia (92.5%). Severe dysplasia was significantly more frequent in the patients with non-homogeneous lesions (5.3%; *p* = 0.00).

With regard to smoking habit, non-homogeneous lesions were significantly more common among non-smokers (65.8%; *p*=0.02). However, no statistically significant differences were observed on evaluating the association between clinical presentation and alcohol (*p* = 0.58).

Table 3 shows the risk factors corresponding to malignant transformation of the lesions. Only four factors were found to be significantly associated to increased transformation risk: non-smokers (*p*=0.001), non-homogeneous lesions (*p*=0.00), lesion location in the tongue (*p* ≤ 0.01) and the presence of epithelial dysplasia (*p* ≤ 0.01).

**Table 3:**
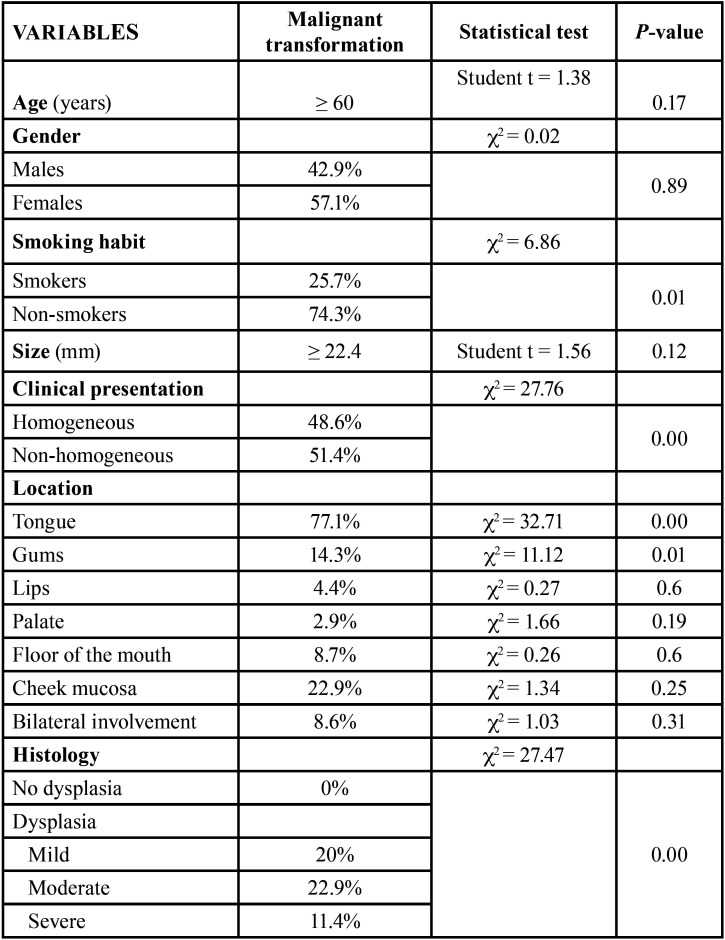
Malignant transformation risk factors.

## Discussion

The present study included 412 patients with the clinical and histopathological features defining oral leukoplakia. The mean patient age at lesion onset was 56.93 years, which is consistent with the observations of authors such as Silverman and Gorsky (12), and Schepman *et al.* (13). Likewise, and in concordance with the data published by Napier *et al.* (14), women were found to be more affected than men. However, most studies in the literature describe a greater prevalence among males. The female predominance seen in our series thus could constitute a limitation on establishing comparisons with other studies.

Smoking is known to be the main etiological factor in OL (10). However, in our series most of the patients (53.2%) were non-smokers. This observation could be related to the fact that most of our patients were women – and smoking is largely associated to the male gender. Although regular alcohol intake is also considered to be a risk factor (15), most of our patients were not regular consumers of alcohol. On the other hand, smoking is considered to possibly influence the clinical presentation and location of the lesions (8). In this regard, and in agreement with the observations of Vladimirov *et al.* (16), we recorded a significant association between non-smoker status and the presence of clinically non-homogeneous lesions.

Most patients with leukoplakia are unaware of the presence of these lesions in their oral cavity. On examining the main reasons for visiting the specialist, we found most of the patients (79.4%) to have been referred by a healthcare professional, thus reflecting the importance of the physician or dentist in establishing the diagnosis of the disease. The great majority of our patients (88.3%) reported no symptoms. This evidences that OL is usually asymptomatic, and that the development of pain or discomfort may be associated to the presence of malignant transformation (17).

Clinically, leukoplakia usually manifests as a homogeneous lesion. In a study on the prevalence of OL in the United States, Scheifele *et al.* (18) found homogeneous lesions to clearly predominate (86.8%) over non-homogeneous lesions (13.2%). Our own findings are consistent with the data reported in the literature, since 81.6% of the patients presented clinically homogeneous lesions versus 18.4% with non-homogeneous lesions.

Histologically, most of the OL lesions in our series of 412 patients showed no epithelial dysplasia (65.7%). Among the lesions exhibiting dysplasia, we found mild dysplasia to predominate (23.8%) – this being consistent with the observations of different authors Vázquez-Alvarez *et al.* (19).

With regard to the association between the clinical manifestations of the lesions and the histopathological findings, the literature describes that homogeneous lesions generally do not exhibit epithelial dysplasia, while non-homogeneous lesions are associated to high grades of dysplasia (14). In our series, practically none of the homogeneous lesions presented epithelial dysplasia (92.5%); furthermore, the percentage of lesions with moderate dysplasia (18.4%) and severe dysplasia (5.3%) was significantly greater in the non-homogeneous OL lesions.

Van der Waal (2) proposed a classification and staging system considering lesion size and histopathological features. In our series, most of the patients presented grade L1P0 lesions, corresponding to stage 1 disease. This is consistent with the results of the study published by Starzyńska *et al.* (20), though Brouns *et al.* (10) found stage 3 to be the most frequent presentation in their patients.

There is currently no consensus regarding the best treatment strategy for patients with OL (7). The main objective is to avoid malignant transformation (21), though management is difficult, since most lesions are refractory to treatment and the relapse rate is high (9). Most of the patients in our series (82.3%) underwent conventional surgery. Brouns *et al.* (22) likewise indicated surgery in most of their patients. Although there is no evidence that any concrete management strategy is truly able to prevent the possible future development of oral squamous cell carcinoma (23), it seems safer to treat all lesions independently of the type of OL involved (7). The decision not to provide treatment should not be regarded as an option, due to ethical reasons (23). In turn, patients should be subjected to follow-up in order to identify any possible changes (21).

Most of the lesions in our series cured or showed no changes after treatment. Nevertheless, 8.5% of the lesions exhibited malignant transformation to oral squamous cell carcinoma. Gándara-Vila *et al.* (24) reported a very similar percentage (8.2%) after an average of 5.58 years of follow-up among their patients with OL. Einhorn and Wersall (25) studied 782 patients with a mean duration of follow-up of 11.7 years, and recorded a 3.93% malignization rate. Pindborg *et al.* (26) obtained very similar results (3.7%), while Roed-Petersen (27) recorded comparatively lower Figures (2.7%).

Six percent of the 670 patients studied by Banoczy (28) showed malignant transformation – this being in line with the results of Warnakulasuriya *et al.* (6.9%) (29) (Table 4).

**Table 4:**
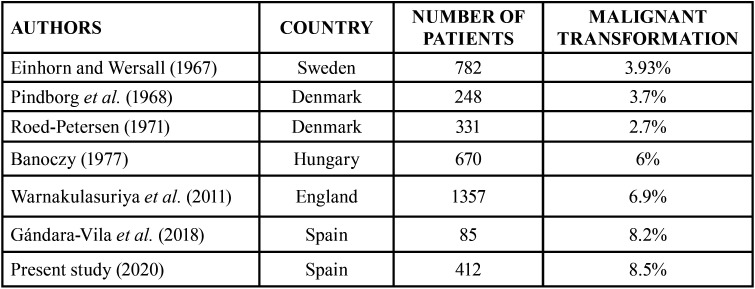
Malignant transformation of oral leukoplakia lesions according to different studies.

No fully reliable individual predictor of malignant transformation has been established to date (7,10). In our series, non-homogeneous lesions were associated to a significant increase in malignization risk. This is consistent with the findings of Gándara-Vila *et al.* (24), who reported a 5-fold greater risk of malignant transformation in the case of non-homogeneous lesions versus homogeneous lesions.

Brouns and van der Waal (22) did not find lesion location to be an indicator of malignization risk. However, in our series, tongue lesions were seen to be significantly associated to malignization, in line with the observations of other authors (1,5,19,29).

The presence and severity of epithelial dysplasia is one of the most important predictors of malignant transformation in OL (1,29,30,). In coincidence with most other investigators, we found the presence and grade of dysplasia to significantly increase the risk of malignant transformation.

Thus, on the basis of the results obtained in our study, it can be concluded that the malignization risk factors are non-smoker status, a non-homogeneous clinical presentation of the lesions, tongue lesions, and the presence and severity of epithelial dysplasia.

## References

[1] van der Waal I (2014). Oral potentially malignant
disorders: is malignant transformation predictable
and preventable?. Med Oral Patol Oral Cir Bucal.

[2] Brouns ER, Baart JA, Bloemena E, Karagozoglu H, van der Waal I (2013). The relevance of uniform reporting
in oral leukoplakia: definition, certainty factor
and staging based on experience with 275
patients. Med Oral Patol Oral Cir Bucal.

[3] van der Waal I (2015). Oral leukoplakia, the ongoing
discussion on definition and
terminology. Med Oral Patol Oral Cir Bucal.

[4] Petti S (2003). Pooled estimate of world
leukoplakia prevalence: a systematic
review. Oral Oncol.

[5] Napier SS, Speight PM (2008). Natural history of potentially
malignant oral lesions and conditions: an overview
of the literature. J Oral Pathol Med.

[6] García-Pola Vallejo MJ, García Martín JM (2002). Oral leukoplakia. Aten Primaria.

[7] van der Waal I (2010). Potentially malignant disorders of
the oral and oropharyngeal mucosa; present
concepts of management. Oral Oncol.

[8] Kumar A, Cascarini L, McCaul JA, Kerawala CJ, Coombes D, Godden D (2013). How should we manage oral
leukoplakia?. Br J Oral Maxillofac Surg.

[9] Lodi G, Porter S (2008). Management of potentially malignant
disorders: evidence and critique. J Oral Pathol Med.

[10] van der Waal I (2009). Potentially malignant disorders of
the oral and oropharyngeal mucosa; terminology,
classification and present concepts of
management. Oral Oncol.

[11] Warnakulasuriya S, Johnson NW, van der Waal I (2007). Nomenclature and classification of
potentially malignant disorders of the oral
mucosa. J Oral Pathol Med.

[12] Silverman SJr, Gorsky M, Lozada F (1984). Oral leukoplakia and malignant
transformation. A follow-up study of 257
patients. Cancer.

[13] Schepman KP, Bezemer PD, van der Meij EH, Smeele LE, van der Waal I (2001). Tobacco usage in relation to the
anatomical site of oral
leukoplakia. Oral Dis.

[14] Napier SS, Cowan CG, Gregg TA, Stevenson M, Lamey PJ, Toner PG (2003). Potentially malignant oral lesions
in Northern Ireland: size (extent)
matters. Oral Dis.

[15] Maserejian NN, Giovannucci E, Rosner B, Zavras A, Joshipura K (2006). Prospective study of fruits and
vegetables and risk of oral premalignant lesions
in men. Am J Epidemiol.

[16] Vladimirov BS, Schiodt M (2009). The effect of quitting smoking on
the risk of unfavorable events after surgical
treatment of oral potentially malignant
lesions. Int J Oral Maxillofac Surg.

[17] Haya-Fernández MC, Bagán JV, Murillo-Cortés J, Poveda-Roda R, Calabuig C (2004). The prevalence of oral leukoplakia
in 138 patients with oral squamous cell
carcinoma. Oral Dis.

[18] Scheifele C, Reichart PA, Dietrich T (2003). Low prevalence of oral leukoplakia
in a representative sample of the US
population. Oral Oncol.

[19] Vázquez-Álvarez R, Fernández-González F, Gándara-Vila P, Reboiras- López D, García-García A, Gándara-Rey JM (2010). Correlation between clini- cal and
pathologic diagnosis in oral leukoplakia in 54
patients. Med Oral Patol Oral Cir Bucal.

[20] Starzyńska A, Pawłowska A, Renkielska D, Michajłowski I, Sobjanek M, Błażewicz I (2014). Oral premalignant lesions:
epidemiological and clinical analysis in the
northern Polish population. Postȩpy Dermatol Alergol.

[21] Holmstrup P, Vedtofte P, Reibel J, Stoltze K (2006). Long-term treatment outcome of oral
premalignant lesions. Oral Oncol.

[22] Brouns E, Baart J, Karagozoglu Kh, Aartman I, Bloemena E, van der Waal I (2014). Malignant transformation of oral
leukoplakia in a well-defined cohort of 144
patients. Oral Dis.

[23] Neville BW, Day TA (2002). Oral cancer and precancerous
lesions. CA Cancer J Clin.

[24] Gandara-Vila P, Perez-Sayans M, Suarez-Penaranda JM, Gallas-Torreira M, Somoza-Martin J, Reboiras-Lopez MD (2018). Survival study of leukoplakia
malignant transformation in a region of northern
Spain. Med Oral Patol Oral Cir Bucal.

[25] Einhorn J, Wersall J (1967). Incidence of oral carcinoma in
patients with leukoplakia of the oral
mucosa. Cancer.

[26] Pindborg JJ, Renstrup G, Jolst O, Roed-Peterson B (1968). Studies in oral leukoplakia: A
preliminary report on the period prevalence of
malignant transformation in leukoplakia based in a
follow-up study of 248 patients. J Am Dent Assoc.

[27] Roed-Petersen B (1971). Cancer development in oral
leukoplakia follow-up of 331
patients. J Dent Res.

[28] Banoczy J (1977). Follow-up studies in oral
leukoplakia. J Max Fac Surg.

[29] Warnakulasuriya S, Kovacevic T, Madden P, Coupland VH, Sperandio M, Odell E (2011). Factors predicting malignant
transformation in oral potentially malignant
disorders among patients accrued over a 10-year
period in South East England. J Oral Pathol Med.

[30] Reibel J (2003). Prognosis of oral pre-malignant
lesions: significance of clinical,
histopathological, and molecular biological
characteristics. Crit Rev Oral Biol Med.

