# Correction: Oral leukoplakia, a clinical-histopathological study in 412 patients

**DOI:** 10.4317/jced.5122335667796

**Published:** 2021-11-01

The article by Rubert and colleagues (*J Clin Exp Dent* 2021;13:426-32, 1 May), is not a retraction, this is a correction of the article. 

